# *Sarcoglycan A* mutation in miniature dachshund dogs causes limb-girdle muscular dystrophy 2D

**DOI:** 10.1186/s13395-020-00257-y

**Published:** 2021-01-07

**Authors:** James R. Mickelson, Katie M. Minor, Ling T. Guo, Steven G. Friedenberg, Jonah N. Cullen, Amanda Ciavarella, Lydia E. Hambrook, Karen M. Brenner, Sarah E. Helmond, Stanley L. Marks, G. Diane Shelton

**Affiliations:** 1 Department of Veterinary and Biomedical Sciences, College of Veterinary Medicine, University of Minnesota, Saint Paul, MN 55113 USA; 2 Department of Pathology, School of Medicine, University of California San Diego, La Jolla, CA 92093-0709 USA; 3 Department of Veterinary Clinical Sciences, College of Veterinary Medicine, University of Minnesota, Saint Paul, MN 55113 USA; 4 Advanced Vetcare, Kensington, Victoria Australia; 5 Centre for Animal Referral and Emergency, Collingwood, Victoria Australia; 6 Animal Referral Hospital, Homebush, New South Wales Australia; 7 Department of Medicine and Epidemiology, School of Veterinary Medicine, University of California, Davis, CA USA

**Keywords:** Canine, Genetics, Myopathy, Sarcoglycanopathy, Gene mutation

## Abstract

**Background:**

A cohort of related miniature dachshund dogs with exercise intolerance, stiff gait, dysphagia, myoglobinuria, and markedly elevated serum creatine kinase activities were identified.

**Methods:**

Muscle biopsy histopathology, immunofluorescence microscopy, and western blotting were combined to identify the specific pathologic phenotype of the myopathy, and whole genome SNP array genotype data and whole genome sequencing were combined to determine its genetic basis.

**Results:**

Muscle biopsies were dystrophic. Sarcoglycanopathy, a form of limb-girdle muscular dystrophy, was suspected based on immunostaining and western blotting, where α, β, and γ-sarcoglycan were all absent or reduced. Genetic mapping and whole genome sequencing identified a premature stop codon mutation in the *sarcoglycan A subunit* gene (*SGCA*). Affected dachshunds were confirmed on several continents.

**Conclusions:**

This first *SGCA* mutation found in dogs adds to the literature of genetic bases of canine muscular dystrophies and their usefulness as comparative models of human disease.

## Background

Muscular dystrophies are a heterogenous group of hereditary degenerative myopathies with variable inheritance patterns. In people, the most common forms include the X-linked Duchenne and Becker muscular dystrophies associated with mutations in the dystrophin gene (*DMD*), and the limb-girdle muscular dystrophies (LGMD) associated with autosomal dominant (LGMD1) or autosomal recessive (LGMD2) mutations in several genes [1, 2]. Further sub-classification of LGMDs is based on an alphabetical system following the order of discovery of their genetic loci. The sarcoglycan complex is part of the dystrophin-glycoprotein complex, composed of four heavily glycosylated glycoproteins (α, β, γ, and δ-sarcoglycan) which help maintain sarcolemmal integrity [3]. Disorders (also termed sarcoglycanopathies) resulting from mutated genes encoding the sarcoglycan-sarcospan complex disrupt sarcolemmal integrity and cause LGMD types 2C-2F, respectively.

Mutations in the dystrophin gene (*DMD*) result in a wide range of clinical severity in human patients, including the milder Becker type MD (BMD) to the severe Duchenne type MD (DMD). Similarly, patients with LGMD may have a wide range of clinical phenotypes including both the severe DMD and milder BMD types with onset from childhood to young adult. A phenotype with exercise intolerance, recurrent rhabdomyolysis, and myoglobinuria has also been described [4, 5]. The serum creatine kinase (CK) activities are usually mildly to markedly elevated. Muscle biopsies from most sarcoglycanopathy patients show dystrophic features. Immunoreactivity for the mutated sarcoglycan subunit is often reduced or absent, but mutations in one sarcoglycan often result in decreased or absence of the other sarcoglycans as well as dystrophin [6]. Thus, it is difficult to reach a diagnosis of a specific sarcoglycanopathy based on immunoreactivity alone.

Several well-described forms of muscular dystrophy are found in domestic dogs, with at least fifteen *DMD* mutations found in over 10 breeds [7–18], as well as two *sarcoglycan delta subunit* (*SGCD*) mutations [19], reported to cause dystrophic clinical phenotypes similar to DMD, BMD, and LGMD, respectively, as described in people. The *DMD* mutation responsible for X-linked muscular dystrophy in golden retriever dogs has been a model for testing new therapeutics for Duchenne type MD for many years [20, 21]. We report here the discovery of the first mutation in the canine *sarcoglycan alpha subunit* (*SGCA*) gene in young adult miniature dachshunds that leads to a form of LGMD with clinical signs that include subclinical myopathy with hyperCKemia, exercise intolerance, progressive weakness, variable dysphagia and pneumonia, and myoglobinuria.

## Methods

### Animals

**Fig. 1:**
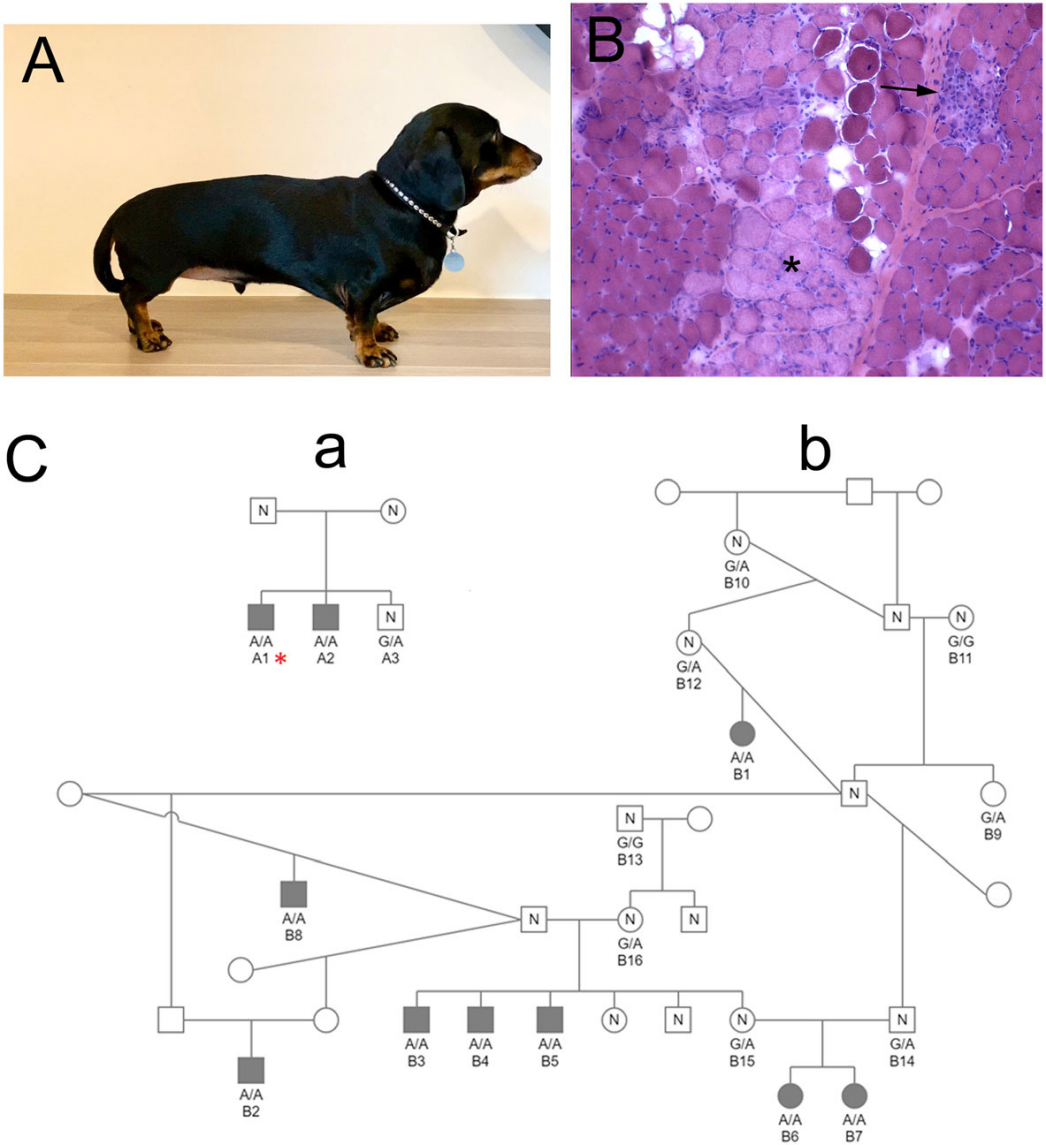
Miniature dachshund dystrophic phenotype and pedigrees. **A** A miniature dachshund from Australia that was evaluated for persistently elevated creatine kinase activity and exercise intolerance. **B** Representative H&E stained cryosection from an affected dachshund showed degenerative (asterisk) and regenerative (arrow) changes consistent with a dystrophic phenotype. **C** One small and one large family that formed the primary basis for this report are presented. Males are designated as squares and females as circles. Cases are solid symbols, controls are open symbols labeled with an “N,” and dogs with unknown phenotypes are open symbols. The case (dog A1) with whole genome sequence data is indicated with an asterisk. Genotypes for the functional *SGCA* variant are provided for all dogs with available samples

The initial diagnostic muscle biopsies or stained cryosections from four young miniature dachshund dogs were submitted by veterinary specialists in Australia to the Comparative Neuromuscular Laboratory, University of California San Diego. The veterinarians reported that all four dogs had persistently elevated CK activities and a myopathic clinical phenotype varying from generalized weakness and exercise intolerance with myoglobinuria to pharyngeal dysphagia (Fig. 1). Additional familial information revealed relationships in which one of the initial four affected dachshunds came from one small pedigree (dog A1, Fig. 1), and three came from a large multi-generational pedigree with extensive interbreeding (dogs B1, B2, and B3, Fig. 1).

Further investigation found six additional affected dogs which could be placed in the pedigrees. One (dog A2) was a sib to the original affected dog of pedigree A, while the five others (dogs B4, B5 B6, B7, and B8) could be placed in pedigree B, often as sibs to other affected dogs. Many parents and unaffected siblings were also identified. All parents were unaffected, and when tested, CK activities from the parents and unaffected siblings were within the reference ranges.

Muscle biopsies from two other dogs, originating in California and South Africa, respectively, were submitted to the Comparative Neuromuscular Laboratory. Both dogs also had markedly elevated CK activities, and clinical signs similar to the initial Australian dogs.

### Histopathology, immunofluorescence microscopy, and western blotting

Biopsies from the biceps femoris, vastus lateralis, and triceps muscles were collected from 6 affected dogs under general inhalational anesthesia by an open biopsy procedure. The biopsy specimens were either unfixed and shipped under refrigeration to the Comparative Neuromuscular Laboratory by an express mailing service or were processed by a local laboratory that shipped the stained cryosections to the Neuromuscular Laboratory for evaluation. Upon receipt, unfixed biopsies were snap-frozen in isopentane pre-cooled in liquid nitrogen and stored at − 80 °C until further processed. In addition, biopsies were immersion fixed in 10% buffered formalin and processed into paraffin. Cryosections (8 μm) were stained or reacted with a standard panel of histological and histochemical stains and reactions [22].

Additional cryosections (8 μm) were cut and stained for indirect immunofluorescence as previously described [23]. Sections were incubated with mouse monoclonal antibodies against the rod (1:100, NCL-DYS1, Novocastra Laboratory) and carboxy terminus (1:100, NCL-DYS2, Novocastra Laboratories) of dystrophin, β-sarcoglycan (1:100, NCL-βSG, Novocastra Laboratories), γ-sarcoglycan (1:100, γSG, Novocastra Laboratories), utrophin (1:20,NCL-DRP2, Novocastra Laboratories), and developmental myosin heavy chain (1:20, NCL-MHCd, Novocastra Laboratories), or with rabbit polyclonal antibodies against laminin α2 (1:200; [24]) and α-sarcoglycan (1:200; [25]). Stainings were visualized using goat anti-rabbit HRP (1:20,000, Invitrogen #31460) or goat anti-mouse HRP (1:20,000, Jackson Immune Laboratory #115-035-003) by previously described immunofluorescent methods [23].

Western blotting was performed by standards methods using extracts from the triceps muscle of an affected dog with a confirmed mutation in α-sarcoglycan (starred dog in Fig. 1) or from archived cryopreserved canine control triceps muscle. Protein bands were separated using NuPage Bis-Tris 4–12% gradient gels (Invitrogen). Primary antibodies included a rabbit polyclonal antibody against α-sarcoglycan (1:2000; [25]) and monoclonal antibodies against β-sarcoglycan (1:1000), γ-sarcoglycan (1:1000), and against β-actin (1:2000, Sigma A2066) as a loading control. Secondary antibodies included peroxidase-conjugated goat anti-mouse IgG (1:20,000, Jackson ImmunoResearch Lab, 115-035-062) and peroxidase-conjugated goat anti-rabbit IgG (1:20,000, Thermo Scientific, 31460). Protein bands were detected using Super Signal West Dura Extended Duration Substrate (Thermo Scientific).

### Genomic data collection and analysis

Eight cases and four unaffected dogs originating from Australia, and the case originating from California, were genotyped on the Illumina CanineHD BeadChip 230 K array. Fourteen dachshund controls collected from unrelated projects conducted at the University of Minnesota were genotyped on the Axiom Canine HD Array 710 K. The SNP genotype data was imputed using a cosmopolitan reference panel of 49 wolves and 2,871 dogs from 183 breeds, including 292 dachshunds genotyped at 710,000 SNPs using Beagle 4.1 [26]. Five hundred ten thousand and nine SNPs remained after pruning the target and reference panels to remove SNPs with more than 2 alleles, minor allele frequency less than 0.001, and discordant genotypes > 0.05 within a multi-breed panel of 360 dogs genotyped at both 230 K and 710 K. PIHAT values for SNPs across the genome obtained from PLINK 1.9 were used to estimate the proportion of identity by descent between dogs with unavailable pedigree information [27]. Haplotypes derived from the imputed data were used to narrow the genomic region flanking a positional and biological candidate gene.

### Whole genome sequencing

A PCR-free library was prepared from a case from pedigree A and sequenced in one lane of an Illumina HiSeq 4000 sequencer by GeneWiz (South Plainfield, NJ 07080). Briefly, a fragment library with an average insert size of ∼ 680 bp was prepared from which ∼ 125 million 2 × 150 bp paired-end reads were generated, corresponding to roughly 17× genome-wide coverage. The reads were mapped against the dog reference genome assembly (CanFam3.1) as described [28, 29] and are available in NCBI’s Short Read Archive (Accession number SRR12537602). Variants identified in the critical interval from the SNP genotype data in the case were compared to those of control genomes from the University of Minnesota’s private WGS database containing 289 dogs of 45 diverse breeds (including 10 dachshunds from unrelated projects). Databases containing variants identified in WGS of more than 1300 dogs of 126 diverse breeds [30, 31] were subsequently searched for the presence of an identified mutation.

### Genotyping assay

The primers used in PCR amplification of the *SGCA* segment containing the reported mutation were 5′-CGTGTCTTTGTGCACACCTT-3′ and 5′-GGGGACTGAGATACCCACAA-3′. The 410 bp amplicon was submitted for Sanger sequencing and the resultant sequence was analyzed for the presence or absence of the mutation with Sequencher 5.1 software (Gene Codes Corporation, Ann Arbor, MI).

## Results

### Clinical findings

Muscle biopsies were received from four Australian miniature dachshunds (3 male and 1 female) ranging in age from 7 to 17 months. Placement of these dogs (A1, B1, B2, B3) within the two pedigrees of Fig. 1 is described in the “Methods” section. No clinical signs of myopathy were apparent in two of the dogs, but markedly elevated CK activities (10,000 to 174,041 IU/L; reference 200–400 IU/L) were noted incidentally on pre-anesthetic blood work. On retesting, the CK activities were persistently elevated. Another affected dog presented for myoglobinuria and weakness after a long walk. The CK activity was markedly elevated (785,000 IU/L). Myoglobinuria resolved within a few days but the CK activity was persistently elevated. One month after presentation, the owner reported chronic poor exercise tolerance. The fourth affected dog was presented for evaluation of chronic pneumonia and surgical correction of a porto-azygous shunt. Pre-anesthetic blood work showed a CK activity of 40,828 IU/L that decreased to 18,309 IU/L following surgical correction of the shunt. Muscle biopsies from all four dogs showed degenerative and regenerative changes consistent with a muscular dystrophy (Fig. 1B). Five additional dogs within the pedigrees of Fig. 1 had persistently and markedly elevated CK activities but tissue was not available for biopsy confirmation of a dystrophic phenotype. The CK activities, where available from unaffected littermates and parents, were within the reference range and no clinical signs of weakness described.

Tissue samples from two additional affected miniature dachshunds, both female and approximately 1 year of age, were subsequently obtained from California and South Africa. One female presented for chronic dysphagia and the other for exercise intolerance and dysphagia. Both dogs had markedly elevated CK activities and a dystrophic phenotype on muscle biopsies.

### Histopathology, immunofluorescence microscopy, and western blotting

**Fig. 2:**
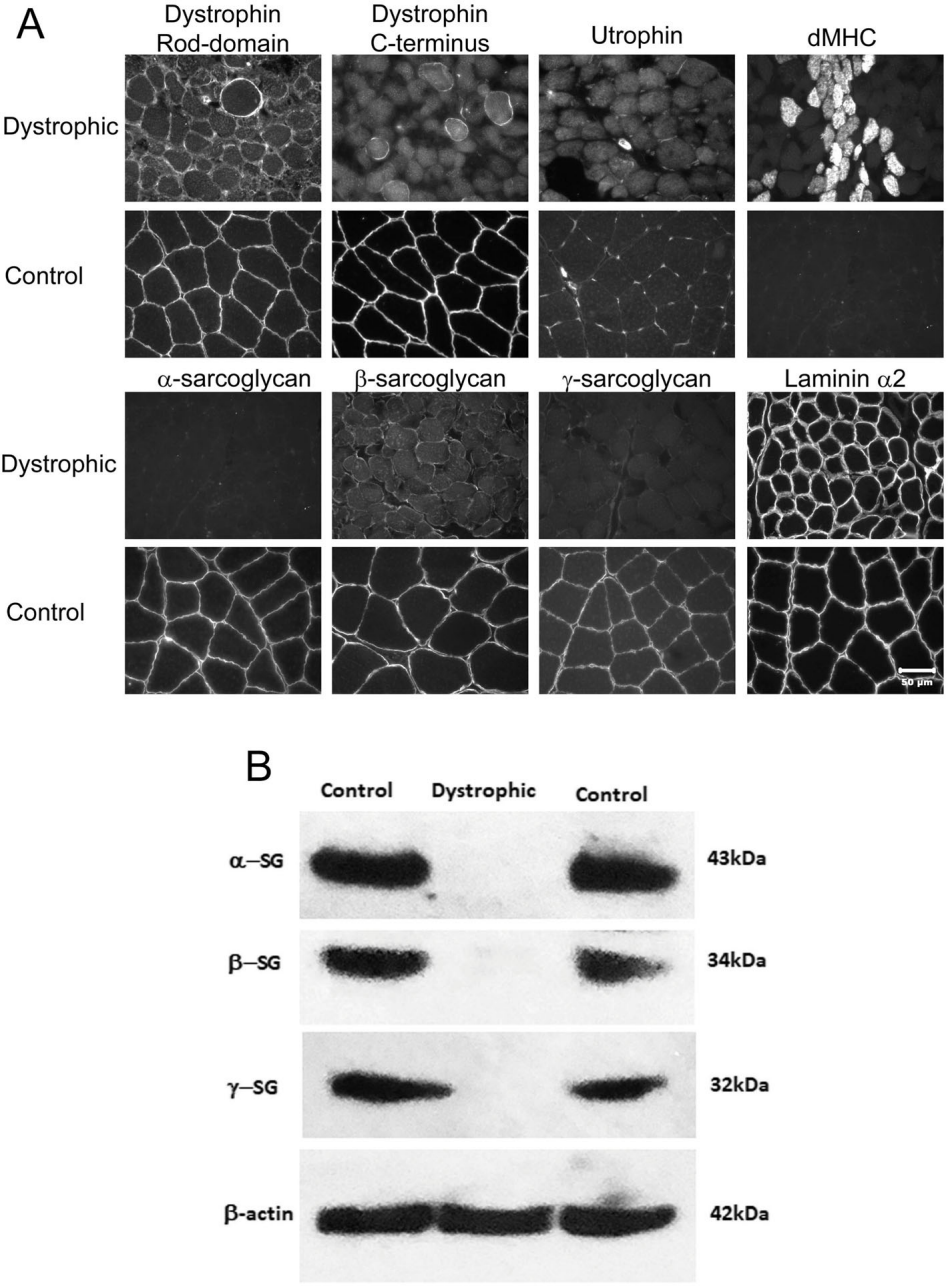
Immunofluorescent staining and western blotting from a dystrophic miniature dachshund. **A** Immunofluorescent staining of muscle cryosections from a representative dystrophic miniature dachshund. Staining for the rod-domain of dystrophin and laminin α-2 was similar to control muscle while staining for the dystrophin C-terminus was patchy. Utrophin was not increased. Numerous regenerating myofibers were highlighted with the antibody against developmental myosin heavy chain (dMHC). Staining for α- and γ-sarcoglycans was absent and decreased for β-sarcoglycan. **B** Western blotting confirmed all three sarcoglycans were absent. β-actin was used as loading control

Pathological changes in muscle biopsies from the four affected miniature dachshunds were dystrophic in nature regardless of the clinical presentation (Fig. 1B). Immunofluorescent antibody staining (Fig. 2A) showed a normal pattern for the dystrophin rod domain and patchy staining with the antibody against the c-terminus of dystrophin. Staining for utrophin and laminin α-2 was similar to control muscle. Clusters of regenerating fibers were highlighted with the antibody against developmental myosin heavy chain (dMHC). Staining for α-sarcoglycan and γ-sarcoglycan was absent and staining was decreased for β-sarcoglycan. A sarcoglycanopathy was suspected based on the staining results. Western blotting (Fig. 2B) confirmed absent staining of protein bands for α, β, and γ-sarcoglycans.

### Genomic analysis

Possible relationships between the pedigrees of Fig. 1C and other dachshunds with SNP array genotyping data were estimated via PIHAT calculations (based on allele sharing across the genome). The average PIHAT in the 27 dog cohort was 0.08. This analysis indicated that the two affected dogs from family A do not have a significant close relationship to dachshunds in family B (PIHAT < 0.05). In addition, the additional case from California had no significant relationships to the established pedigrees (PIHAT < 0.05).

As an autosomal recessive trait was suspected, we first used a genotypic analysis model in which all nine cases were required to be homozygous for the minor allele, while controls could have all three genotypes. 444 SNPs met the criteria, with 112 of them lying in an ~ 19 Mb segment on chromosome 9 between bp positions 18,928,814–37,617,735. When these 444 SNPs were ranked by *p* value, 18 of the top 25 SNPs and 35 of the top 50 most significant SNPs were in a ~ 12-Mb segment of chromosome 9 (bp positions 18,958,229 to 30,966,212), with *p* values ranging from 9.4 × 10^−5^ to 2.1 × 10^−7^) (Table 1).

**Table 1:** Top 50 SNPs in a genotypic model

CHR	SNP	A1	A2	AFF	UNAFF	*P*
9	9:25951885	C	T	9/0/0	0/8/10	2.134e−07
9	9:25983462	C	G	9/0/0	0/7/11	2.134e−07
9	9:26288724	C	T	9/0/0	0/6/12	2.134e−07
9	9:26292362	G	A	9/0/0	0/6/12	2.134e−07
9	9:26334051	A	G	9/0/0	0/6/12	2.134e−07
9	9:26345821	T	C	9/0/0	0/7/11	2.134e−07
9	9:26347986	C	T	9/0/0	0/8/10	2.134e−07
34	34:10784553	G	A	9/0/0	0/12/6	2.134e−07
34	34:10786591	C	A	9/0/0	0/12/6	2.134e−07
9	9:25213000	T	C	9/0/0	1/6/11	2.134e−06
9	9:26313263	C	T	9/0/0	1/6/11	2.134e−06
18	18:39450835	G	A	9/0/0	1/11/6	2.134e−06
9	9:24972813	C	G	9/0/0	1/7/10	4.267e−06
9	9:24974987	G	A	9/0/0	1/7/10	4.267e−06
9	9:24994921	T	G	9/0/0	1/7/10	4.267e−06
9	9:25040066	G	C	9/0/0	1/7/10	4.267e−06
9	9:25046429	T	G	9/0/0	1/10/7	4.267e−06
9	9:25049565	G	A	9/0/0	1/10/7	4.267e−06
9	9:26020165	A	G	9/0/0	1/10/7	4.267e−06
9	9:26398124	C	T	9/0/0	1/8/9	6.401e−06
18	18:39447278	C	T	9/0/0	1/9/8	6.401e−06
9	9:25373707	C	T	9/0/0	2/4/12	1.174e−05
4	4:27820959	T	C	9/0/0	2/10/6	1.387e−05
4	4:27831539	T	C	9/0/0	2/10/6	1.387e−05
4	4:27868268	A	G	9/0/0	2/10/6	1.387e−05
9	9:30966212	C	T	9/0/0	2/6/10	1.387e−05
34	34:10891254	G	A	9/0/0	2/10/6	1.387e−05
34	34:10893260	A	C	9/0/0	2/10/6	1.387e−05
9	9:19304983	C	T	9/0/0	2/8/8	1.984e−05
9	9:26289840	A	G	9/0/0	2/8/8	1.984e−05
32	32:10752374	T	C	9/0/0	2/8/8	1.984e−05
1	1:106899476	A	G	9/0/0	2/11/5	2.347e−05
9	9:25394028	C	G	9/0/0	2/11/5	2.347e−05
9	9:24966631	C	G	9/0/0	2/7/9	3.136e−05
9	9:25053412	A	G	9/0/0	2/9/7	3.136e−05
9	9:25065255	T	C	9/0/0	2/9/7	3.136e−05
9	9:25444400	T	C	9/0/0	2/7/9	3.136e−05
9	9:25493635	A	G	9/0/0	2/7/9	3.136e−05
34	34:10909969	C	A	9/0/0	2/9/7	3.136e−05
4	4:26209208	C	T	9/0/0	3/4/11	5.868e−05
6	6:28794412	G	T	9/0/0	3/11/4	5.868e−05
4	4:26426217	A	G	9/0/0	3/5/10	9.388e-05
6	6:28865921	G	T	9/0/0	3/12/3	9.388e−05
9	9:18958229	G	A	9/0/0	3/10/5	9.388e−05
9	9:19012795	A	T	9/0/0	3/10/5	9.388e−05
9	9:19023729	T	G	9/0/0	3/10/5	9.388e−05
9	9:19122430	A	G	9/0/0	3/10/5	9.388e−05
9	9:19123171	T	G	9/0/0	3/10/5	9.388e−05
9	9:24989017	A	G	9/0/0	3/10/5	9.388e−05
9	9:25380184	A	C	9/0/0	3/5/10	9.388e−05

The table lists the top 50 SNPs in which all nine cases were required to be homozygous, while controls could have all three genotypes. The chromosome (CHR), position (SNP), minor allele (A1), major allele (A2), number of affected dogs homozygous for the minor allele/heterozygous/homozygous for the major allele (AFF), number of unaffected dogs homozygous for the minor allele/heterozygous/homozygous for the major allele (UNAFF), and the *p* value for association (*P*) are provided

A similar result was seen in a straight recessive model, in which all nine cases were required to be homozygous for the minor allele and the controls could be either heterozygous or homozygous for the major allele (Table 2). In this scenario, 32 of the top 50 SNPs were in the same ~ 12 Mb region of chromosome 9 identified above. Further, in either analysis, only nine SNPs (seven on chromosome 9 and two on chromosome 34) met a criterion in which no controls could be homozygous for the minor allele.

**Table 2:** Top 50 SNPs in a recessive model

CHR	SNP	A1	A2	AFF	UNAFF	*P*
9	9:25951885	C	T	9/0	0/18	2.134e−07
9	9:25983462	C	G	9/0	0/18	2.134e−07
9	9:26288724	C	T	9/0	0/18	2.134e−07
9	9:26292362	G	A	9/0	0/18	2.134e−07
9	9:26334051	A	G	9/0	0/18	2.134e−07
9	9:26345821	T	C	9/0	0/18	2.134e−07
9	9:26347986	C	T	9/0	0/18	2.134e−07
34	34:10784553	G	A	9/0	0/18	2.134e−07
34	34:10786591	C	A	9/0	0/18	2.134e−07
9	9:24972813	C	G	9/0	1/17	2.134e−06
9	9:24974987	G	A	9/0	1/17	2.134e−06
9	9:24994921	T	G	9/0	1/17	2.134e−06
9	9:25040066	G	C	9/0	1/17	2.134e−06
9	9:25046429	T	G	9/0	1/17	2.134e−06
9	9:25049565	G	A	9/0	1/17	2.134e−06
9	9:25213000	T	C	9/0	1/17	2.134e−06
9	9:26020165	A	G	9/0	1/17	2.134e−06
9	9:26313263	C	T	9/0	1/17	2.134e−06
9	9:26398124	C	T	9/0	1/17	2.134e−06
18	18:39447278	C	T	9/0	1/17	2.134e−06
18	18:39450835	G	A	9/0	1/17	2.134e−06
1	1:106899476	A	G	9/0	2/16	1.174e−05
4	4:27820959	T	C	9/0	2/16	1.174e−05
4	4:27831539	T	C	9/0	2/16	1.174e−05
4	4:27868268	A	G	9/0	2/16	1.174e−05
9	9:19304983	C	T	9/0	2/16	1.174e−05
9	9:24966631	C	G	9/0	2/16	1.174e−05
9	9:25053412	A	G	9/0	2/16	1.174e−05
9	9:25065255	T	C	9/0	2/16	1.174e−05
9	9:25373707	C	T	9/0	2/16	1.174e−05
9	9:25394028	C	G	9/0	2/16	1.174e−05
9	9:25444400	T	C	9/0	2/16	1.174e−05
9	9:25493635	A	G	9/0	2/16	1.174e−05
9	9:26289840	A	G	9/0	2/16	1.174e−05
9	9:30966212	C	T	9/0	2/16	1.174e−05
32	32:10752374	T	C	9/0	2/16	1.174e−05
34	34:10891254	G	A	9/0	2/16	1.174e−05
34	34:10893260	A	C	9/0	2/16	1.174e−05
34	34:10909969	C	A	9/0	2/16	1.174e−05
1	1:120378888	C	T	9/0	3/15	4.694e−05
4	4:26209208	C	T	9/0	3/15	4.694e−05
4	4:26216500	A	G	9/0	3/15	4.694e−05
4	4:26426217	A	G	9/0	3/15	4.694e−05
6	6:28794412	G	T	9/0	3/15	4.694e−05
6	6:28865921	G	T	9/0	3/15	4.694e−05
6	6:70411742	T	A	9/0	3/15	4.694e−05
9	9:18928814	C	T	9/0	3/15	4.694e−05
9	9:18958229	G	A	9/0	3/15	4.694e−05
9	9:19012795	A	T	9/0	3/15	4.694e−05
9	9:19023729	T	G	9/0	3/15	4.694e−05

The table lists SNPs in which all nine cases were required to be homozygous for the minor allele and the controls could be either heterozygous or homozygous for the major allele. The table lists the top 50 SNPs in which all nine cases were required to be homozygous, while controls could have all three genotypes. The chromosome (CHR), position (SNP), minor allele (A1), major allele (A2), number of affected dogs homozygous for the minor allele/heterozygous plus homozygous for the major allele (AFF), number of unaffected dogs homozygous for the minor allele/heterozygous plus homozygous for the major allele (UNAFF), and the *p* value for association (*P*) are provided

**Fig. 3:**
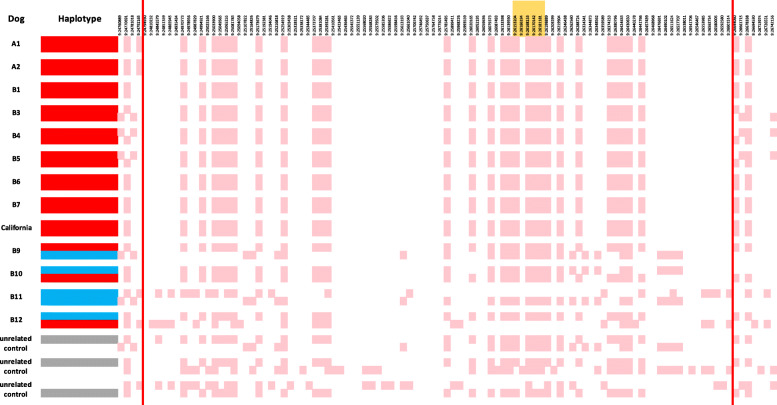
Haplotypes in the region of interest on CFA9. SNP coordinates are provided along the top row. A pink cell indicates the minor allele and a white cell indicates the major allele. Identification numbers on the far left refer to the dogs within the pedigrees of Fig. 1. The CFA9 haplotype in which all 9 cases were homozygous spanned from 24,792,165–26,644,060 Mb. The affected haplotype is denoted with a red highlight (left side), unaffected haplotypes have a blue highlight, and gray haplotypes are identical to the affected haplotype but do not have the *SGCA* c.G224A mutation. The *SGCA* gene, located from positions (26,164,863–26,174,864 Mb), is indicated by an orange bar along the top

Phasing of the SNP genotype data from this chromosome 9 segment revealed a haplotype in which all 9 cases were homozygous (Fig. 3), where a minimally-conserved ~ 1.8-Mb haplotype ranged from CFA9:24,792,165 to 26,644,060 Mb. The figure also shows that the central region of the minimally-conserved haplotype is common across miniature dachshunds. A high frequency of many of the alleles comprising the affected haplotype exists throughout the control population and it is flanking SNPs that tag the affected haplotype. Fifty-one genes with unique IDs are in this segment, including *sarcoglycan A* (*SGCA*, ENSCAFG00000017013), at position CFA9:26,164,863–9:26,174,864 Mb. No significantly associated SNPs in the vicinity of the genes for other subunits of the sarcoglycan-sarcospan complex (*SGCB*, CFA13:44,972,473–44,987,184; *SGCG*, CFA25:15,249,622–15,430,044; *SGCD*, CFA4:53,263,866–53,820,231; and *SSPN,* CFA27:21.359,164–21,392,382) were identified.

### Whole genome sequencing and mutation identification

Both the human and canine *SGCA* genes have eight protein-encoding exons. Analysis of WGS from the vicinity of *SGCA* in a case (Fig. 1) identified a premature stop codon in exon 3; specifically, CFA9:26,166,312 G > A; c.G224A; p. W75*. This variant was not found in any of the more than 1300 dogs across published WGS databases [29, 30]. The predicted stop codon at residue 75 is expected to truncate 80% of the 387 amino acid sarcoglycan A protein. Interestingly, a flanking haplotype identical to that found in homozygosity in all nine cases was also present in the control population; however, the *SGCA* c.G224A mutation genotype in such control dogs demonstrated the wild type allele in these haplotypes. There were no other homozygous coding variants in *SGCA* and no other unique coding variants in the 1.8-Mb haplotype.

### Pedigree and population screening

*SGCA c.G224A* variant genotypes from the available members of the pedigrees were entirely consistent with autosomal recessive inheritance, with available parents being obligate heterozygotes, and other relatives being heterozygotes or homozygous wild type (Fig. 1C). The two additional dachshund cases from South Africa and California were homozygous for the *SGCA c.G224A* variant as well. One hundred and ten dachshunds available from submissions to the University of Minnesota for unrelated projects were all clear of the mutation.

## Discussion

Here we describe the first canine cases of LGMD2D with a mutation in the α-sarcoglycan gene. In people, age of onset of LGMD2D is reported to be from childhood to young adult and in the miniature dachshunds of this report, onset was from 7 to 17 months of age. Similar to people with LGMD2D [4, 5], the clinical features varied in severity among the affected dogs ranging from subclinical myopathy with hyperCKemia to more generalized weakness and myoglobinuria. Such variability in the severity of clinical signs is not restricted to the LGMDs but has also been reported in humans with DMD [32] and in dogs with XLMD [33]. Similar to the dachshunds of this report, a markedly elevated CK activity was also an incidental finding on pre-anesthetic blood screening prior to neuter from a family of Labrador retrievers with dystrophin-deficient muscular dystrophy [34]. No clinical evidence of myopathy was described by the owners or detected on physical examination. However, muscle biopsies were dystrophic, and dystrophin protein was absent on western blotting and markedly decreased to absent on immunostaining. Detection of a markedly and persistently elevated CK activity should be an indicator of an underlying congenital myopathy in young dogs.

Although a sarcoglycanopathy was suspected based on immunostaining and western blotting, the specific sarcoglycan protein responsible could not be determined, as α, β, and γ-sarcoglycan were all absent or reduced. The dystrophic pattern on the muscle biopsies and the clinical presentations also did not aid in the differentiation of the type of dystrophy. Pedigree analysis suggested an autosomal recessive disorder, so an X-linked form of MD (canine XLMD) was less likely, even though staining of the carboxy terminus of dystrophin was patchy. This patchy staining can be secondary to the sarcoglycan deficiency. While the severity of clinical signs was milder than that described in a previous publication of δ-sarcoglycan deficiency in Boston terrier dogs [19], pathological changes in muscle biopsies and the persistently high CK activity were similar.

Whole genome SNP array genotyping data identified a region on CFA9 containing the *SGCA* gene where all cases were homozygous. A highly probable functional variant in the *SGCA* gene was identified from WGS, where a premature stop codon is predicted to truncate approximately 80% of the protein. A recessive inheritance pattern was confirmed by targeted genotype assays that demonstrated that all affected dogs were homozygous for the variant, unaffected relatives were heterozygous or homozygous for the reference allele, and the unaffected parents were all heterozygous. Although the two identified pedigrees did not have close genetic relationships, affected dachshunds with the identical mutation to that found in the Australian families have now been found in South Africa and California, suggesting dispersal by a common founder. The relatively small 1.8-Mb-affected haplotype further supports a hypothesis that the *SGCA* arose several generations ago.

The canine and human *SGCA* amino acid sequences are both 387 amino acids long and the residues share 90% identity. Fifteen of the 39 amino acid differences between these species are in the first 60 N-terminal residues. Functional protein domains of note are a single transmembrane alpha helix near the C-terminal, 2 asparagine residues in the C-terminal half of the protein that serve as glycosylation sites, and a phosphorylatable serine residue near the C-terminus. Several thousand human *SGCA* variants have been identified and at least 30 are associated with human LGMD (UniProtKB - Q16586; March 1, 2020). These functional variants are spread across residues 30–284, with over half of them in the first third of the sequence. The canine variant database lists 111 *SGCA* variants, but only one of them affects the coding sequence, a missense coding variant with a SIFT score that does not predict damage to the protein structure. In any event, a stop codon mutation that truncates 80% of the SGCA protein is extremely likely to produce a nonfunctional protein even if the mRNA is not degraded and a partial polypeptide is translated.

## Conclusions

This combined clinical, pathological, immunofluorescence, western blotting, and molecular genetic approach has identified a *sarcoglycan A subunit* mutation in miniature dachshund dogs that results in a sarcoglycanopathy, a form of limb-girdle muscular dystrophy. This first *SGCA* mutation found in dogs adds to the literature of genetic bases of canine muscular dystrophies and their usefulness as comparative models of human disease. Further, this demonstrates that persistent CK elevations should not be dismissed in young animals without clinical evidence of myopathy.

## Data Availability

The WGS of the affected dog is available under BioProject title: “Sarcoglycan A mutation in miniature dachshund dogs”. Accession number SRR12537602.

## Abbreviations

CFA9: Canis familiaris chromosome 9
DMD: Duchenne muscular dystrophy
*DMD*: Dystrophin gene
GWAS: Genome-wide association analysis
LGMD: Limb-girdle muscular dystrophy
*SGCA*: Sarcoglycan A subunit
SNP: Single nucleotide polymorphism
WGS: Whole genome sequence
